# *Trichinella spiralis* excretory/secretory products from adult worms inhibit NETosis and regulate the production of cytokines from neutrophils

**DOI:** 10.1186/s13071-023-05979-8

**Published:** 2023-10-20

**Authors:** Jing Wang, Bin Tang, Xihuo You, Xuepeng Cai, Wanzhong Jia, Xiaolei Liu, Mingyuan Liu, Xuemin Jin, Jing Ding

**Affiliations:** 1 State Key Laboratory for Diagnosis and Treatment of Severe Zoonotic Infectious Diseases, Key Laboratory for Zoonosis Research of the Ministry of Education, Institute of Zoonosis, and College of Veterinary Medicine, Jilin University, Changchun, 130062 China; 2 Beijing Agrichina Pharmaceutical Co., Ltd., Wangzhuang Industrial Park, Airport Road, Shahe, Changping District, Beijing, 102200 China; 3 State Key Laboratory of Veterinary Etiological Biology, Key Laboratory of Veterinary Parasitology of Gansu Province, Lanzhou Veterinary Research Institute, Chinese Academy of Agricultural Sciences, Lanzhou, 730046 China

**Keywords:** *Trichinella spiralis*, Excretory and secretory products, Neutrophil extracellular traps, Immune response

## Abstract

Upon encountering exogenous pathogens, polymorphonucleocytes (PMNs) engage in various processes to destroy them, including releasing neutrophil extracellular traps (NETs) that trap pathogens and induce phagocytosis and cytokine production. Parasites have unique strategies with which to evade the host's immune response. However, the strategy employed by *Trichinella spiralis* in response to the reaction of PMNs has yet to be elucidated. This study explored the effect of excretory/secretory products (ESP) on three major functions: NETs, phagocytosis, and cytokine production. Specifically, PMNs were pre-treated with the ESP of 3-day-old adults and then stimulated with phorbol 12-myristate 13-acetate (PMA). We found that in PMNs pretreated with ESP, PMA-induced NET generation was suppressed by ESP. ROS production is a hallmark of PMA-induced NETosis. The LDH assay results showed that ESP inhibits NETs by suppressing ROS rather than promoting PMN death. Furthermore, ESP enhanced *Escherichia coli* engulfment by PMNs, improving overall phagocytic function. Finally, cytokine analysis revealed an increase in pro-inflammatory cytokine IL-1β, and other cytokines (IL-10, TNF-α), while IL-4 displayed a significant reduction. In conclusion, this study has unraveled *T. spiralis*' evasion and regulation mechanisms against innate immune cells, providing insights into parasite strategies to manipulate host immunity, potentially informing new treatments for NET-related autoimmune diseases.

**Graphical Abstract:**

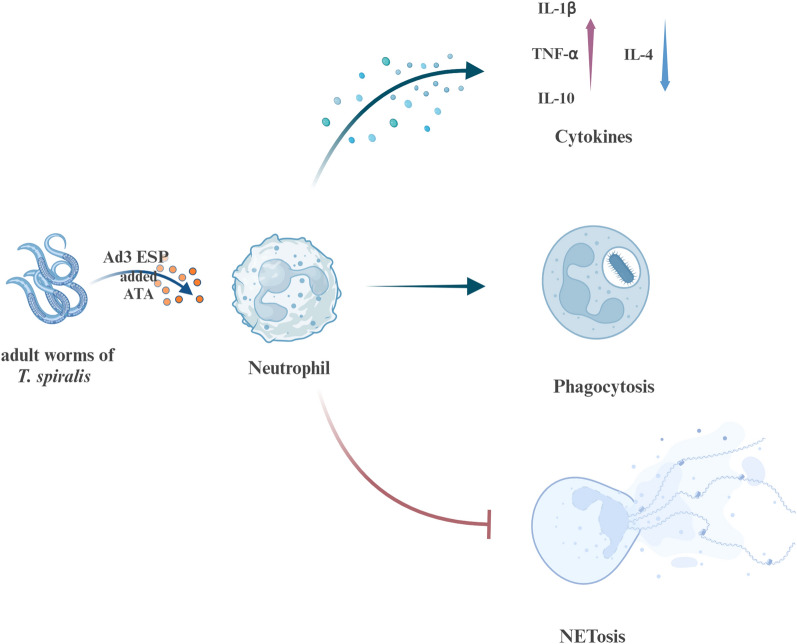

**Supplementary Information:**

The online version contains supplementary material available at 10.1186/s13071-023-05979-8.

## Introduction

Humans are constantly exposed to pathogenic microorganisms that can cause life-threatening infections, and it is the immune system's job to protect against pathogen colonization and infection. Among the immune cells, polymorphonucleocytes (PMNs), a type of granulocyte, play a vital role in the host's defense against intruders. They perform numerous functions, including cytokine release, phagocytosis, and the formation of neutrophil extracellular traps (NETs). In the early stages of microbial infection, PMNs play a crucial role in killing pathogens through several mechanisms, including the production of various cytokines such as TNF-α, IL-1β, and IL-6, releasing granular enzymes, coordination of the immune response, recruitment of other immune cells to the site of infection, and supporting the clearance of the infection [1]. PMNs employ phagocytosis, an essential function to detect, engulf, and destroy microbes, including bacteria, fungi, protozoa, and parasites, using their plasma membrane to form a phagosome that fuses with lysosome-containing enzymes and toxic substances to eliminate the microbe [2–4]. This process promotes the occurrence of killing mechanisms, including reactive oxygen species (ROS), acid pH, and antimicrobial proteins, which ultimately destroy and eliminate the microbe. Furthermore, the generation of ROS by the NADPH oxidase complex is an important part of PMN function. NADPH oxidase activation is linked to NET generation, a novel mechanism of extracellular microbial killing [5]. NET formation occurs in response to a wide range of stimuli, including lipopolysaccharide (LPS), IL-8, and Phorbol-12-myristate-13-acetate (PMA), as well as parasites such as *Toxoplasma gondii* and *Leishmania* [6, 7]. NETosis can be induced in vitro once robust NADPH oxidase has been activated through PMA stimulation [8]. Previous studies have shown that respiratory burst and ROS production are crucial for NET formation [9]. It was previously believed that after NET release, PMN death occurs. More recent studies have shown that PMNs can also release NETs containing mitochondrial, but not nuclear, DNA. In this instance, NET release does not cause cell death, and the cells still retain their complete structure and phagocytic ability [10].

Although PMNs have numerous strategies to eliminate pathogens, invading microbes have also formed various mechanisms to avoid this, including inhibition of NET formation, NET degradation by the secretion of nucleases, and resistance to the damage of the antimicrobial proteins adhering to NETs [11]. Over the past decade, research has shown that these three mechanisms are also employed by pathogenic bacteria, viruses, and parasites. However, it is unknown whether helminth parasites, such as *Trichinella spiralis*, can also evade NETs via one or more of these mechanisms. It is commonly accepted that parasites generally disturb the physiological and immunological homeostasis of the host to establish long-term infection [12]. The first level of parasite-host communication can be simple protein-protein interactions [12], and much attention has therefore been paid to excretory-secretory products (ESP). Although the functional analysis of ESP in parasites has received the most attention, its role in regulating PMN-mediated responses is much less defined, and its function in forming NETs has yet to be fully explored. Recent studies have suggested that certain parasites, such as *Nippostrongylus brasiliensis* [13], *Ostertagia ostertagi* [14], and *Eimeria bovis* [15], can induce the formation of NETs.

This study explored the effect of ESP of adult *T. spiralis* on PMN phagocytosis and PMA-induced NET formation and assessed the cytokine expression of PMNs to determine whether *T. spiralis* ESP is involved in immune regulation by modulating PMN-derived cytokines. Results revealed that the ESP of adult *T. spiralis* significantly reduced the amount of PMA-induced NETs and ROS production, potentially compromising innate immune response in the host and facilitating the establishment of a chronic infection. Furthermore, the study demonstrated that ESP enhances PMN phagocytosis and modulates PMN-derived cytokines. Overall, the findings deepen our understanding of the inhibitory effect of *T. spiralis* ESP on NETs and its effect on cytokine production, which may aid in developing novel therapies aimed at NETs and NET-related autoimmune conditions.

## Materials and methods

### Animals and bacterial strains

Female C57BL/6 mice (6–8 weeks old) and Wistar rats (200 ± 20 g) were purchased from the Experimental Animal Center of the College of Basic Medical Sciences, Jilin University (Changchun, China). All animals were kept in pathogen-free conditions, and all experiments were performed under the guidelines of the Administration of Experimental Animals in China. The protocol was approved by the Institutional Animal Care and Use Committee of Jilin University (20170318).

*Escherichia coli* BL21 (DE3) (Sangon, China) was transformed with PFM23 plasmid (constructed from pET28A, Novagen, USA) to facilitate the cytoplasmic production of enhanced green fluorescent protein (EGFP) under the control of the T7 promoter to analyze bacterial phagocytosis. The EGFP-*E. coli* were maintained and cultured on LB agar supplemented with 50 μg/ml kanamycin at 37 °C in an incubator and stored at 4 °C in a refrigerator for subsequent use.

### Parasite and ESP collection

*Trichinella spiralis* (ISS534) were grown and maintained in our laboratory by serial passage in Wistar rats and isolated as previously described [17]. Infective-stage muscle larvae of *T. spiralis* were recovered from the skeletal muscles of infected Wistar rats at 35 days post-infection (dpi) via artificial digestion of minced muscle in a solution containing 1% pepsin-1% HCl for 2 h at 37 °C with agitation [18]. Adult-stage parasites were isolated from the small intestines of *T. spiralis*-infected Wistar rats at 3 dpi and referred to as Ad3. *Trichinella spiralis* were washed three times in normal saline solution and then maintained in RPMI-1640 medium (Gibco, USA) supplemented with 100 U/ml penicillin and 100 µg/l streptomycin at 37 °C under 5% CO_2_ for 16 h. After incubation, the supernatant containing ESP was concentrated using an Amicon Ultra-3 Centrifugal Filter Unit (molecular weight [MW] cut-off, 3 kDa; Millipore, USA) at 4 °C and 5000*g* for 30 min [19]. The endotoxin levels in ESP (0.002 EU) were determined by Limulus amoebocyte lysate (LAL, Zhanjiang A&C Biological Ltd., Guangdong Province, China); the concentrations of endotoxin in ESP for further use were < 0.005 EU. Protein concentration, i.e., ESP, was determined by a BCA Protein Assay Kit (Beyotime Biotech., China).

### Isolation of mouse bone-marrow-derived PMNs

PMNs were isolated from C57BL/6 as previously described with modifications [20]. Briefly, the mice were killed and soaked in 70% ethanol. Bone marrow cells were collected by syringe flushing the femurs with phosphate-buffered saline (PBS). PMNs were subsequently isolated and purified from the bone marrow cells using a mouse Neutrophil Isolation Kit (P8550, Solarbio, China) according to the manufacturer's instructions. PMNs were collected by centrifugation at 1500 rpm for 10 min, after which they were washed three times and resuspended in ice-cold PBS at a concentration of 5 × 10^5^ cells/ml. Cells were incubated with antibodies LY6G (1:500 dilution; Abcam Inc., USA) and CD11b (1:500 dilution; Abcam Inc., USA) in the dark for 40 min at 4 °C. Then, they were rewashed before analysis by flow cytometry to determine the purity of the PMN isolates. Flow cytometry data were analyzed using FlowJo (Three Star Inc., USA). To confirm nuclear morphology, isolated cells were seeded into 24-well glass-bottomed black plates at 2 × 10^5^ cells/well and subsequently stained with 5 µM Hoechst 33342 (Sigma, USA). Then, the samples were observed under a laser scanning confocal microscope (Olympus FluoView FV1000, Olympus America, Inc., USA).

### Cell viability

A Cell-Counting Kit 8 (CCK8; Beyotime Biotechnology, China) was used to test relative cell viability according to the manufacturer’s instructions. Briefly, PMNs were seeded in 96-well plates (2 × 10^3^ cells/well), which were divided into ATA-treated groups (containing PMNs with ATA concentrations of 5, 10, 15, 20, 25, and 30 μM), control group, and blank group with five holes in each group. The control group contained PMNs and medium, and the blank group only contained medium. After 3 h incubation, 10 μl CCK-8 solution was added and incubated in the incubator for 1 h. The absorbance at 450 nm was determined in a multimode microplate reader (Thermo Fisher Scientific, USA).

### Visualization and quantification of NETs

PMNs were seeded into 24-well glass-bottomed black plates at 4 × 10^5^ cells/well and then incubated at 37 °C with 5% CO_2_. Each experiment included three groups: blank control group, experimental group, and negative control group. Previous work has shown endonuclease activity in *T. spiralis* ESP [21]. To eliminate DNase activity in the ESP, 25 μM nuclease inhibitor aurintricarboxylic acid (ATA) was added along with different concentrations of Ad3 ESP (2.5, 5, and 10 ng/μl) to the experimental group, and PMNs were pre-treated for 1 h. PMNs were then stimulated with 100 nM PMA for 3 h. After incubation, PMNs were stained with 5 µM Hoechst 33342 (Sigma, USA) and 5 µM SYTOX Green (Invitrogen, USA), and the samples were observed under a laser scanning confocal microscope (Olympus FluoView FV1000, Olympus America, Inc., USA). In addition, alterations in PMN morphology and nuclear dimensions were assessed by a laser scanning confocal microscope (Olympus FluoView FV1000, Olympus America, Inc., USA), and the nuclear expansion area for 500 cells in each sample well across the respective groups was quantified using ImageJ software (National Institutes of Health, USA).

### ROS detection

The intracellular formation of ROS by activated PMNs was detected using the oxidation sensitive dye 2′, 7 dichlorofluorescein diacetate (DCFH-DA) (Sigma, USA) as the substrate. In the presence of intracellular ROS, DCFH-DA is oxidized to form highly fluorescent dichlorofluorescein (DCF). PMNs were seeded into 96-well cell culture plates with black frames at 1 × 10^5^ cells/well. DCFH-DA was added to the cultures to give a final concentration of 10 µM. Samples were incubated at 37 °C with 5% CO_2_ for 20 min; the plates were then washed twice with phenol red-free medium, and different concentrations of Ad3 ESP (1, 2, 5, and 10 ng/μl) were added to each well. The experimental and control groups were then stimulated with 100 nM PMA for 3 h at 37 °C with 5% CO_2_. The fluorescence plate reader (Tecan Infinite F200) was used to detect fluorescence at an excitation of 485 nm and an emission of 535 nm.

### LDH detection

Lactate dehydrogenase (LDH) released from PMA-treated PMNs was quantified to determine whether NET reduction was due to ESP cytotoxicity. Briefly, PMNs were inoculated into a 96-well cell culture plate at 1 × 10^5^ cells/well, and different concentrations of Ad3 ESP (2, 5, and 10 ng/μl) with 25 μM ATA were added into the culture medium. After 1 h incubation, PMNs were stimulated with 100 nM PMA for 3 h. All steps were maintained at 37 °C with 5% CO_2_. The plate was centrifuged at 400*g* for 5 min, and 120 μl supernatant from each well was transferred to a new 96-well plate. The LDH activity in the supernatants was determined using an LDH Cytotoxicity Assay Kit (Roche Applied Science, Germany) according to the manufacturer's protocol after incubation in darkness for 30 min. Samples included the experimental group, a background control, negative control of unstimulated PMN (0% cell lysis), and positive control of maximum LDH release (100% cell lysis).

### Phagocytosis assay

Neutrophil phagocytosis was evaluated using the following two experimental methods: First, the phagocytic capacity of PMNs was quantitatively assessed by measuring the phagocytic index using the Vybrant Phagocytosis Assay Kit (Thermo Fisher Scientific, USA) following the manufacturer's instructions. Briefly, PMNs (1 × 10^5^ cells/well in 96-well plates) were treated or untreated with ESP at different concentrations (1, 2, 5, and 10 ng/µl) and incubated at 37 °C for 2 h. After incubation, the supernatants were replaced with 100 µl fluorescent particles (in 100 µl PBS), provided by the Vybrant Phagocytosis Assay Kit, and further incubated at 37 °C for 2 h in the dark. The reaction was terminated by adding 100 μl of ice-cold trypan blue suspension. Phagocytosis was quantified by measuring the fluorescence of each sample using a microplate-reading fluorimeter at an excitation of 494 nm and an emission of 518 nm (Biotek, USA). The percentage of phagocytosis was calculated using the following formula: % effect (phagocytosis) = (absorbance value of experimental group − background absorbance)/(absorbance value of positive group − absorbance value of negative group) × 100%.

The second method involved three groups: a blank control group (containing only PMNs), a negative control group (with the addition of 10 ng/µl ESP only), and an experimental group (with the addition of 25 µM ATA and 10 ng/µl ESP). After the pretreatment, each group (5 × 10^5^ cells/well in 24-well plates) was combined with EGFP-*E. coli* (initial concentration of 1 × 10^7^ CFU/ml) and incubated at 37 °C for 2 h, with the multiplicity of infection (MOI) adjusted to MOI 25. Next, the PMNs co-cultured with EGFP-*E. coli* were centrifuged at 2500 rpm for 10 min to remove the suspended EGFP-*E. coli* and obtain PMNs. After resuspension in PBS, Hoechst 33342 (1:1000) dye was added, and the samples were observed using a laser scanning confocal microscope (Olympus FluoView FV1000, Olympus America, Inc., USA). The supernatants from each group were collected and centrifuged at 12,000 rpm for 1 min. The obtained pellets were resuspended in pre-chilled PBS and then serially diluted at a ratio of 10. Aliquots of 100 µl from each dilution were spread onto LB agar plates and then inverted and incubated at 37 °C or 14–18 h. The number of colonies at each dilution was counted to determine the CFU/ml, corresponding to the decrease in the number of viable bacteria in the supernatant, which was an indirect measure of phagocytosis.

### Quantitative PCR (qPCR)

Total RNA from PMNs treated or untreated with Ad3 ESP was extracted using an RNAprep Pure Cell Kit (TianGen, China) according to the manufacturer's instructions. cDNA was produced using a Thermo Scientific RevertAid First Strand cDNA Synthesis Kit (Thermo Scientific, USA) according to the manufacturer's recommendations for oligo (dT) primed cDNA synthesis performed on 1 μg RNA. Finally, qPCR was performed using the SYBR Green qPCR Master Mix (TaKaRa, Japan) according to the manufacturer's instructions. Primers used for qPCR are listed in Table 1. All pairs of F/R primers were synthesized by Sangon Biotechnology Inc. qPCR was performed with a reaction mixture with a total volume of 25 μl: 12.5 μl SYBR Green qPCR Master Mix (2 ×), 1 μl forward primer (10 μmol/l), 1 μl reverse primer (10 μmol/l), 2 μl template, and 8.5 μl ddH_2_O. The qPCR profile used is as follows: initial denaturation at 95 °C for 5 min, followed by amplification of 40 cycles at 95 °C for 15 s, 56 °C for 30 s, and 72 °C for 15 s. All steps were performed using a Step One Plus Real Time PCR System (Applied Biosystems, USA). The relative mRNA levels of the target genes were calculated by 2^−ΔΔCT^.

**Table 1:** Primers used in the RT-qPCR

Genes	Primer sequence (5′ → 3′)		Length (bp)	Accession No
IL-1β	F:	TTCAGGCAGGCAGTATCAC TCATTG	169	NM_031512.2
	R:	ACACCAGCAGGTTATCATCATCATCC		
IFN-γ	F:	CTCTTCTTGGATATCTGGAGGAACTGG	138	NM_138880.2
	R:	AATGACGCTTATGTTGTTGCTGATGG		
IL-10	F:	TTCTTTCAAACAAAGGACCAGC	81	NM_012854.2
	R:	GCAACCCAAGTAACCCTTAAAG		
IL-4	F:	TACCAGGAGCCATATCCACGGATG	139	NM_201270.1
	R:	TGTGGTGTTCTTCGTTGCTGTGAG		
TNF-α	F:	GCGACGTGGAACTGGCAGAAG	92	NM_012675.3
	R:	GAATGAGAAGAGGCTGAGACATAGGC		
GAPDH	F:	CAAGTTCAACGGCACAGTCA	100	NM_017008.4
	R:	CCATTTGATGTTAGCGGGAT		

The nucleotide sequences of the qPCR primers used to assay gene expression by real-time quantitative PCR are shown

### ELISA analysis of cytokine expression

PMNs were incubated with or without Ad3 ESP (10 ng/μl) for 3 h, and supernatants were harvested for cytokine analysis. IL-1β, IFN-γ, IL-10, IL-4, and TNF-α levels were measured using the corresponding mouse ELISA kits (Invitrogen, USA) according to the manufacturer's instructions.

### Statistical analysis

All experiments were conducted in quintuplicate (*n* = 5) or sextuplicate (*n* = 6) and repeated three times. All results are expressed as mean ± SEM. Statistical analysis was performed using GraphPad Prism 5.0 software (GraphPad Software, USA). One-way analysis of variance (ANOVA) was used to compare differences between different conditions. *P* < 0.05 was considered statistically significant. *P* values are expressed as *P < 0.05, **P < 0.01, ***P < 0.001.

## Results

### Ad3 ESP inhibits PMA-induced NET formation

**Fig. 1:**
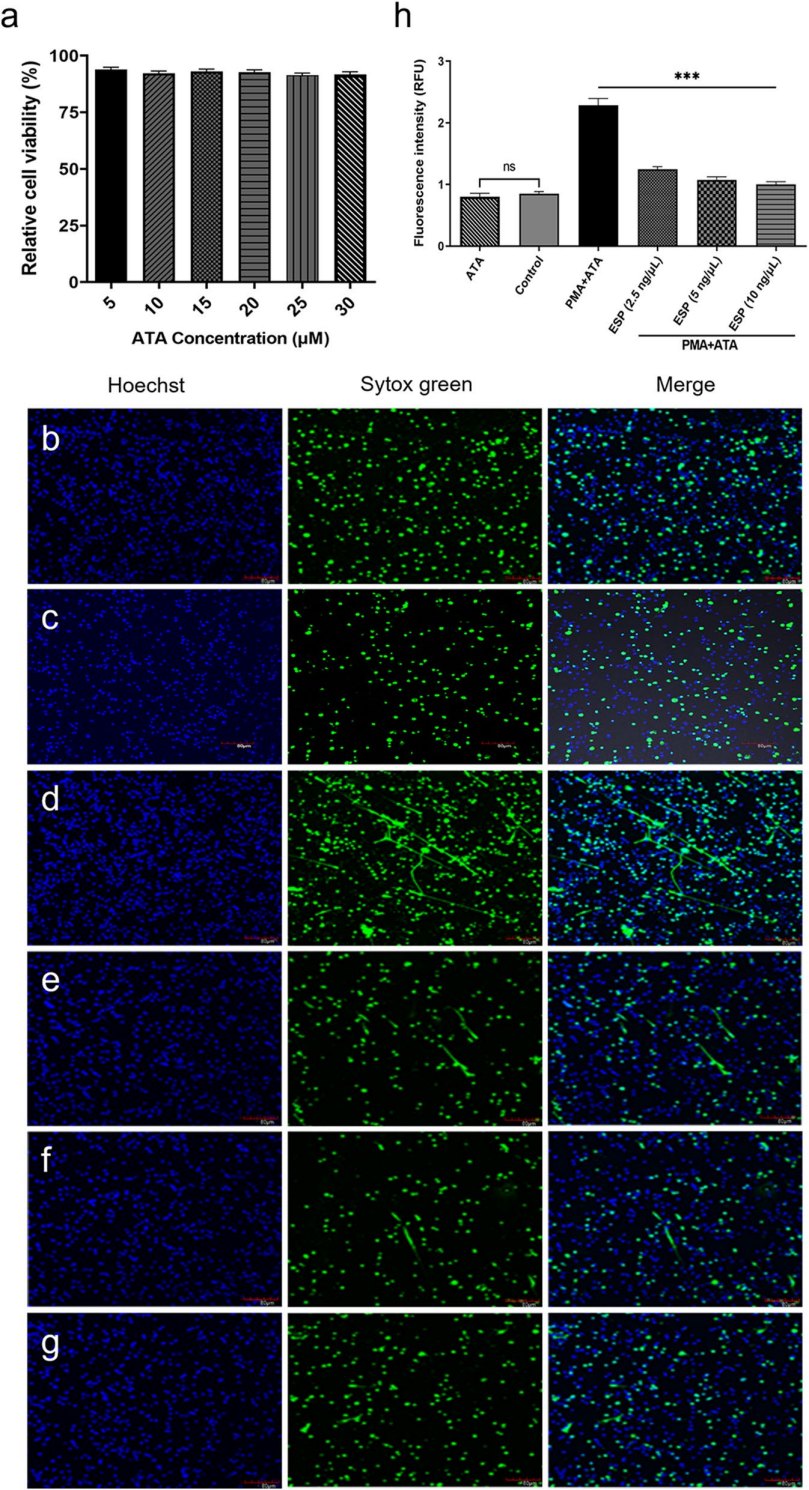
Effect of ATA on neutrophils cell activity. **a** The cell viability of neutrophils was assessed under different ATA concentration treatments (5, 10, 15, 20, 25, and 30 μM) using the CCK-8 assay (*n* = 5). Different concentrations of ESP impede PMA-stimulated NET formation. **b** Unstimulated neutrophils were used as a control group. **c** Neutrophils were incubated with 25 μM ATA alone. **d** Neutrophils were treated with 100 nM PMA and 25 μM ATA. **e**–**g** Neutrophils were pretreated with Ad3 ESP at different concentrations (2.5, 5, and 10 ng/μl) containing 25 μM ATA for 1 h at 37 °C before incubation with PMA. NETs were visualized by staining with Hoechst 33342 (blue) and Sytox Green (green). Merged representative images of at least three experiments are shown. Scale bars = 80 μm. **h** Extracellular DNA quantification by fluorometry. ***P < 0.001. ATA, aurintricarboxylic acid; CCK-8, Cell-Counting Kit 8; ESP, excretory/secretory products; PMA, phorbol-12-myristate-13-acetate. NETs, neutrophil extracellular traps

**Fig. 2:**
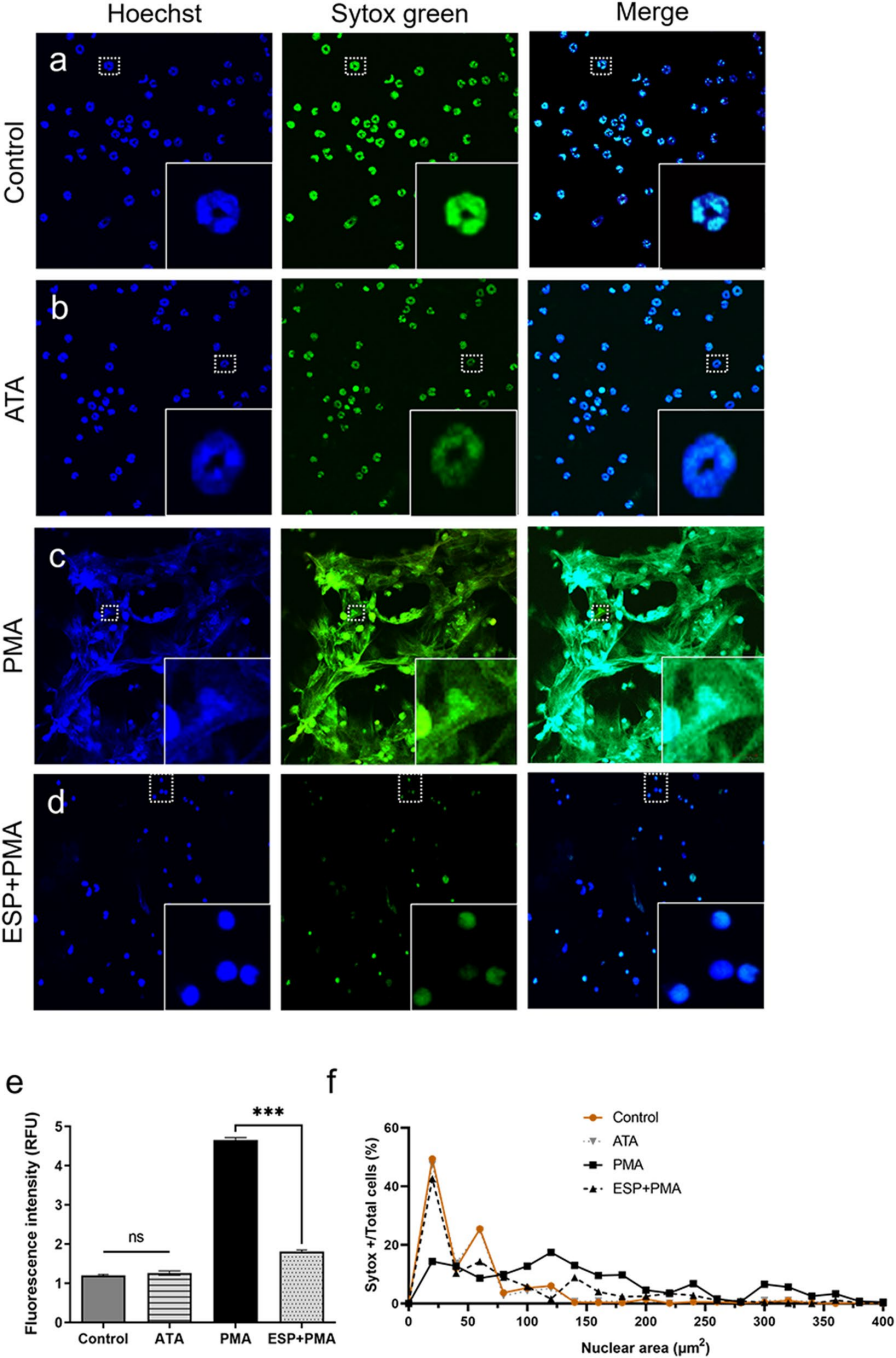
Neutrophils pre-treated with excretory/secretory products exhibit reduced NET formation and altered nuclear morphology. The formation of NETs is reduced, accompanied by alterations in nuclear morphology. **a**, **b** The nuclear morphology of neutrophils (4 × 10^5^) was untreated (**a**) or treated with 25 μM ATA alone (**b**). **c**, **d** 4 × 10^5^ neutrophils were stimulated with 100 nM PMA for 3 h to induce NET formation or preincubated with 10 ng/μl ESP (added ATA) for 1 h before stimulation with PMA. Neutrophils were stained with Sytox green (green, extracellular DNA) and Hoechst 33342 (blue, total DNA) dyes (magnification 20×). The boxes show a close-up of the area indicated with white dotted lines. **e** Extracellular DNA quantification by fluorometry. ***P < 0.001. **f** Nuclear area quantification was carried out using ImageJ software. ATA, aurintricarboxylic acid; ESP, excretory/secretory products; PMA, phorbol-12-myristate-13-acetate. NETs, neutrophil extracellular traps

PMNs were purified to the required level (Additional file 1: Fig. S1) for further experimentation using a previously described method [20]. Notably, the cell activity in each group exceeded 90% (Fig. 1a), indicating that the concentrations (25 μM) used in this experiment did not induce cellular damage. PMNs were pre-incubated with different concentrations of Ad3 ESP containing 25 μM ATA and subsequently stimulated with 100 nM PMA for 3 h. The NETs of each group were observed under a laser confocal microscope (Fig. 1b–g). Unstimulated PMNs and ATA alone did not show NETs (Fig. 1b, c). Figure 1d shows the structure of the abundant NETs released by PMNs incubated with PMA + ATA. The NET formation was significantly reduced in the presence of different ESP (added ATA) concentrations (2.5, 5, and 10 ng/μl) (Fig. 1e–g). NET production nearly ceased at 10 ng/μl concentration of Ad3 ESP, leading to an observable and quantifiable reduction in NETs (Figs. 1d–g, 2d). The nuclear morphology of the pre-treated PMNs was altered, with nuclei appearing diffused and having indistinct boundaries (Fig. 2c), as opposed to the characteristic lobulated nuclear shape observed in untreated cells and ATA alone (Fig. 2a, b). Consequently, a concentration of 10 ng/μl ESP (Fig. 2) was employed in the subsequent experiments. To corroborate this observation, we quantified nuclear expansion and found that the nuclei of PMNs pre-incubated with ESP (added ATA) were slightly enlarged compared to untreated PMNs, but they did not reach the same level as those in PMA-stimulated PMNs (Figs. 1, 2e). These results were consistent with the quantitative assessment of nuclear expansion, demonstrating that pre-treatment with ESP significantly reduced nuclear expansion (Fig. 2e, f).

### NET inhibition was due to ESP reducing ROS production and not ESP cytotoxicity

**Fig. 3:**
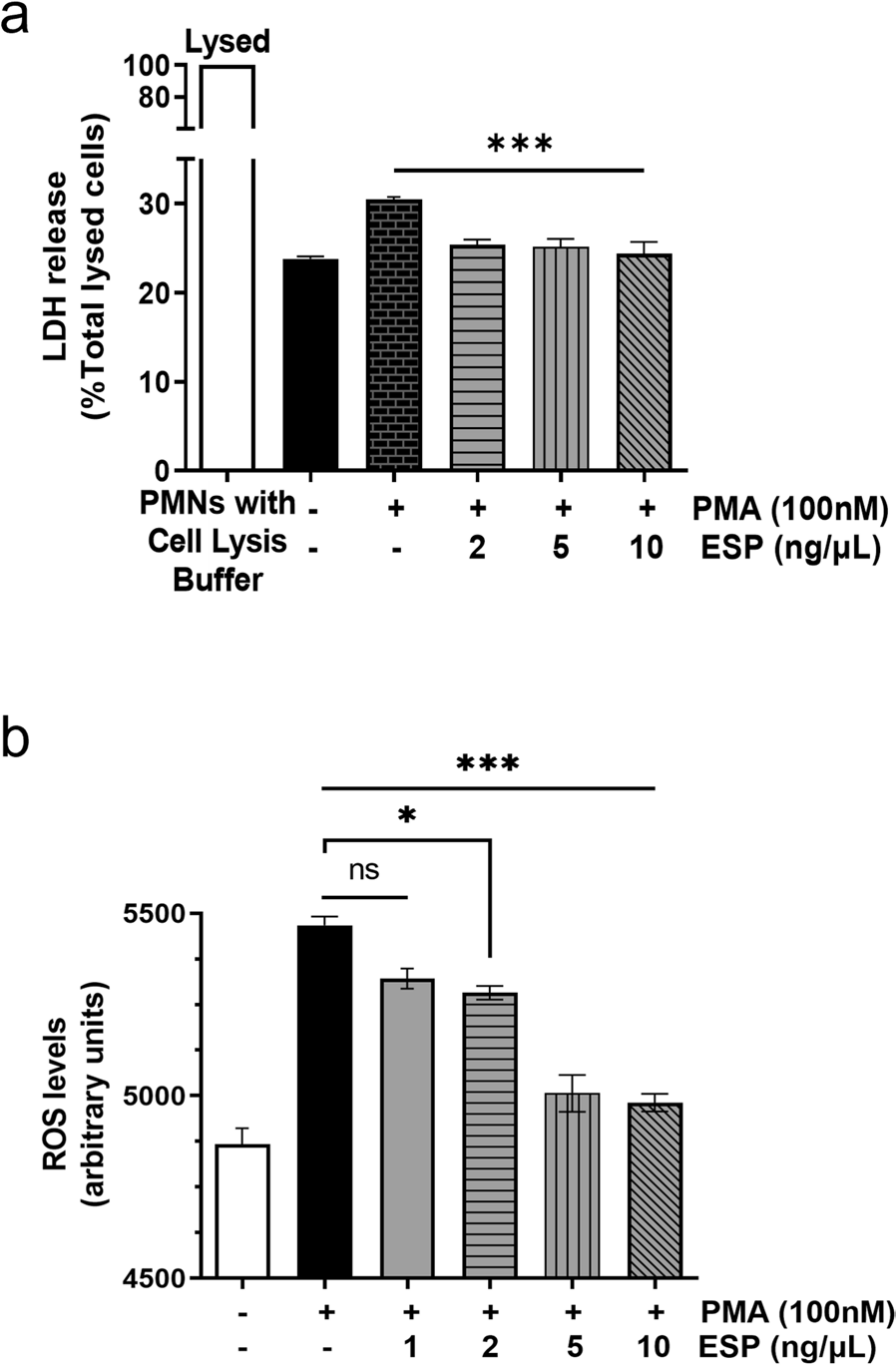
Excretory/secretory products suppress the production of NETs due to the decrease in the generation of ROS. **a** Neutrophils were untreated or pre-treated with various concentrations of ESP (added ATA) (2, 5, 10 ng/μl) for 1 h at 37 °C, followed by stimulation with PMA (100 nM) for 3 h at 37 °C. LDH released from damaged cells is normalized to the total LDH content of an equivalent number of neutrophils lysed with detergent (complete lysis). LDH levels were significantly lower in the ESP-pretreated group compared to the PMA-stimulated group. The white column represents complete lysis (100%) of neutrophils with cell lysis buffer, while the black column indicates untreated neutrophils. ***P < 0.001. **b** Neutrophils pre-treated with different concentrations of ESP (added ATA) (1, 2, 5, 10 ng/μl, 1 h, 37 °C) prior to PMA activation (100 nM, 3 h, 37 °C). ROS production in neutrophils was measured by the luminol-amplified chemiluminescence method. ROS levels were significantly reduced in ESP-treated neutrophils compared with the PMA-stimulated group. All data are expressed as mean (± standard error mean, SEM) (N for both experimental and control groups = 5); *P < 0.05; ***P < 0.001. ESP, excretory/secretory products; ATA, aurintricarboxylic acid; PMA, phorbol-12-myristate-13-acetate. LDH, lactate dehydrogenase; NETs, neutrophil extracellular traps; ROS, reactive oxygen species

LDH activity assessment in the PMN-conditioned medium, after pretreatment with Ad3 ESP and subsequent PMA stimulation, revealed that the diminished NET release was not associated with increased PMN death but was speculated to be related to the production of ROS (Fig. 3). No significant inhibition of LDH release was observed in PMNs pre-incubated with ESP (added ATA) at concentrations of 2 and 5 ng/μl. However, the LDH activity was significantly decreased at 10 ng/μl ESP (added ATA) (***P < 0.001) (Fig. 3a). To substantiate our hypothesis that reduced NET release is associated with decreased ROS production, we evaluated ROS generation in PMNs exposed to PMA (100 nM) alone or combined with Ad3 ESP (added ATA) pretreatment. Results revealed that the ESP pre-treated samples exhibited lower ROS levels compared to PMA-stimulated samples (Fig. 3b). As expected, a significant (*P < 0.05, ***P < 0.001) inhibitory effect on ROS production in response to PMA was observed with pretreatment of up to 2 ng/μl Ad3 ESP (added ATA). This observation suggests that the inhibition of NETs is due to the ability of ESP to decrease ROS production rather than being a result of ESP-induced cytotoxicity.

### Ad3 ESP promotes PMN phagocytosis

**Fig. 4:**
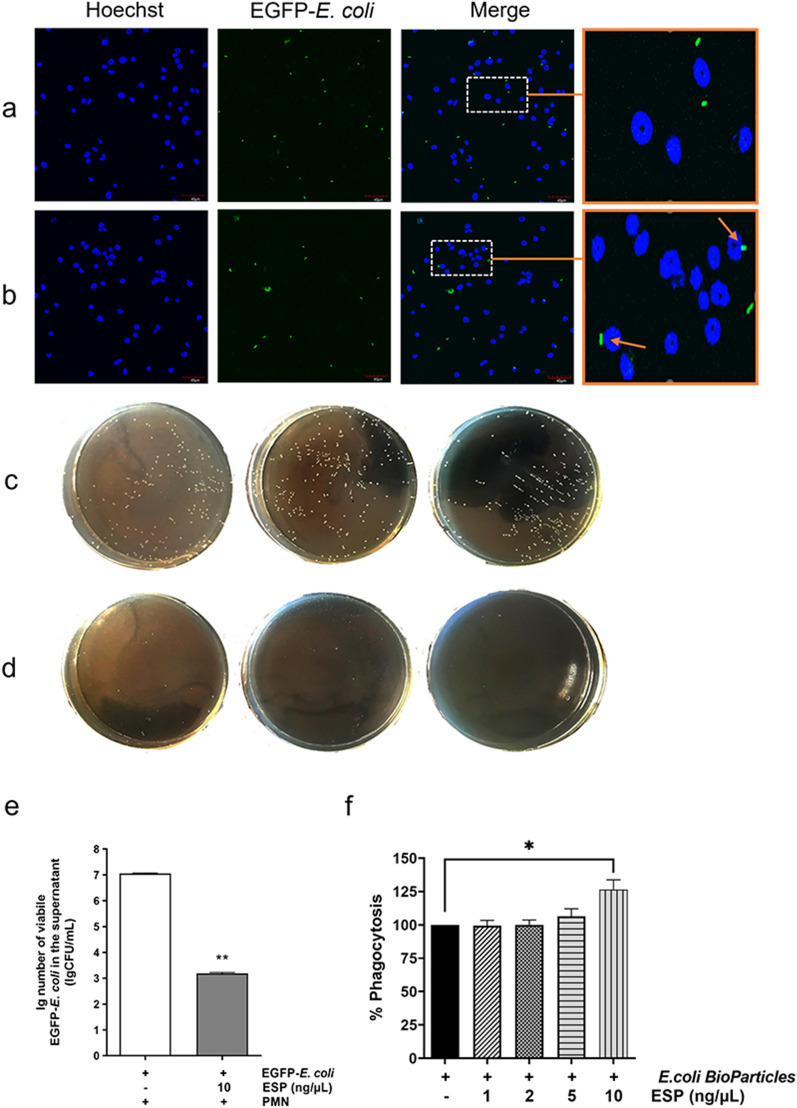
Excretory/secretory products enhanced the phagocytosis activity of neutrophils. **a**, **b** ESP enhanced the phagocytosis of live EGFP-*Escherichia coli* by neutrophils. Neutrophils were infected with EGFP-*E. coli* (MOI 25) (green fluorescence) for 2 h. Higher magnifications of selected phagocytosis areas are shown on the rightmost side. The arrowheads indicate the EGFP-*E. coli* ingested by neutrophils. The bars in the original image represent 40 μM (excluding the rightmost magnifying box). **c**–**e** Representative images of neutrophils untreated (**c**) or pre-treated with 10 ng/μl ESP (added ATA) (**d**) for 2 h, showing increased phagocytic activity after ESP pretreatment. **e** A total of 5 × 10^5^ neutrophils per ml was incubated with 1 × 10^7^ EGFP-*E. coli* per ml for 2 h. After co-incubation, the viable bacterial counts in the supernatant were calculated. Values are means (± standard error mean, SEM) (shown by error bars) of at least three independent experiments. **P < 0.01. **f** Neutrophils, either pre-treated or untreated, were incubated with bioparticles from Vybrant Phagocytosis Assay Kit for 2 h in the dark. The phagocytic index of neutrophils untreated or pre-treated with different concentrations of ESP (added ATA) (1, 2, 5, and 10 ng/μl) for 2 h at 37 °C (means ± standard error mean; *N* = 5; *P < 0.05). ESP, excretory/secretory products; ATA, aurintricarboxylic acid; EGFP, *E. coli*, enhanced green fluorescent protein-*E. coli*

Both direct and indirect methods were applied to determine whether Ad3 ESP affects the phagocytic activity. Direct methods included observation with a laser scanning confocal microscope (Fig. 4a, b) and measurement of the phagocytic index (Fig. 4e). As shown in Fig. 4, when PMNs were exposed to Ad3-ESP (added ATA) at a concentration of 10 ng/μl, the green fluorescence would gather around the blue nucleus stained by Hoechst, and the amount of EGFP-*E. coli* with green fluorescence entering the cytoplasm increased, while the amount in the culture supernatant decreased (Fig. 4b) compared to the untreated group (Fig. 4a). Conversely, indirect methods involved counting the number of EGFP-*E. coli* in the culture supernatant (Fig. 4c–e). PMNs were either pre-incubated with different concentrations of Ad3 ESP (1, 2, 5, 10 ng/μl) or left untreated. Lower concentrations of Ad3 ESP (added ATA) (1, 2, 5 ng/μl) did not affect PMN phagocytosis (Fig. 4f). However, as shown in Fig. 4e, PMNs pre-treated with Ad3 ESP (added ATA) at a concentration of 10 ng/μl (Fig. 4b, d) had a significantly increased phagocytosis capacity compared to untreated PMNs (Fig. 4a, c) (**P < 0.01).

### Ad3 ESP regulates PMN cytokine expression

**Fig. 5:**
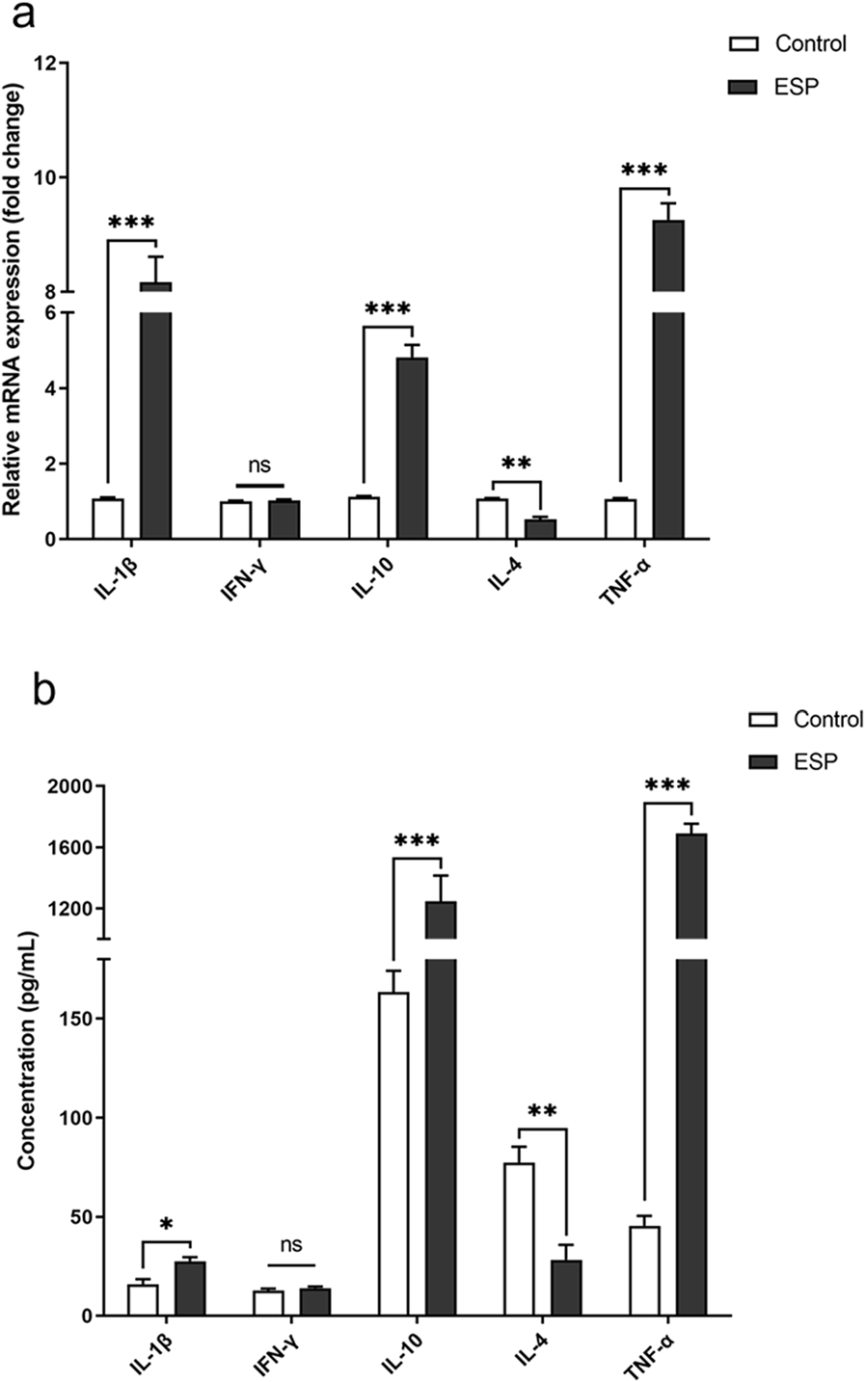
Excretory/secretory products regulate neutrophils’ cytokine secretion. IL-1β, IFN-γ, IL-10, IL-4, and TNF-α mRNA expression and concentrations were analyzed from 10 ng/μl ESP-pretreated neutrophils (solid bars), untreated neutrophils (white bars). **a** qPCR analysis of cytokine mRNA levels. **b** The culture supernatants from two groups were collected after 3 h for cytokine measurement by ELISA. The results represent the mean (± standard error mean, SEM) of three independent experiments. Statistical analysis was performed to compare differences between the control (untreated neutrophils) and ESP-treated group. *P < 0.05; **P < 0.01; ***P < 0.001. ESP, excretory/secretory products

To investigate Ad3 ESP's possible immunomodulatory activity, we selected PMN-derived cytokines, such as IL-1β, IFN-γ, IL-10, IL-4, and TNF-α, based on previous studies [22]. Both methods revealed significant differences in cytokine levels between the two groups depicted in Fig. 5 (qPCR and ELISA). qPCR was used to analyze cytokine mRNA levels in untreated and Ad3 ESP-treated PMNs. The results showed that IL-1β, IL-10, and TNF-α levels were significantly increased (***P < 0.001) and IL-4 significantly decreased (**P < 0.01) (Fig. 5a). ELISA was used to analyze cytokine levels in supernatants. IL-10, TNF-α, and IL-1β levels were significantly increased (*P < 0.05, ***P < 0.001) and IL-4 significantly decreased (**P < 0.01) (Fig. 5b). It was evident that both ELISA and qPCR tests showed no significant differences in the changes of IFN-γ and that the ELISA results were almost consistent with qPCR results.

## Discussion

The fundamental mechanisms by which PMNs eliminate microbes encompass phagocytosis, the secretion of cytokines and chemokines, induction of degranulation, promotion of ROS generation and release, and NET formation [23]. This study found that ESP pre-treated PMNs exhibited enhanced phagocytic capacity; as a result, the number of *E. coli* colonies was significantly reduced. A recent study demonstrated that targeting NET-associated extracellular DNA with DNase treatment promotes bacterial clearance and facilitates phagocytosis, which in turn unmasks bacteria (such as *Streptococcus pneumoniae*) trapped in NETs and exposes them to intact PMNs [24]. In addition, the study revealed that DNase-mediated degradation of NETs generates short DNA fragments that further enhance PMN phagocytosis. However, according to varying regulatory effects of different forms of secretoglobin family 1A member 1 (SCGB 1A1) on the PMN phagocytic function, it has previously been observed that SCGB 1A1A, rather than SCGB 1A1, significantly enhances the phagocytic activity [25]. This further suggests that the compositions of Ad3 ESP and ml ESP are different. Therefore, our findings on the effects of Ad3 ESP on PMN phagocytosis differ from those reported by Ríos-López et al. regarding ml ESP [16]. Moreover, various cytokines produced by PMNs play crucial roles in influencing the immune response and the interaction of immune cells. Therefore, in this study, expression levels of PMN-derived cytokines, including pro-inflammatory (IL-1β and IFN-γ), tumor necrosis factor superfamily members (TNF-α), and other cytokines (IL-4 and IL-10), were detected using qPCR and ELISA, and among all the cytokines examined, TNF-α expression was most notably increased. In this regard, research has suggested that the upregulation of TNF-α is associated with enhanced phagocytic activity in PMNs [26]. Our results also showed a significant increase in PMN-derived IL-1β and IL-10 production. Neutrophil recruitment is regulated by pro-inflammatory cytokines such as IL-1β [27]. We demonstrated that Ad3 ESP strongly stimulated PMNs to secrete IL-1β, thereby regulating their recruitment to inflammatory sites. Moreover, it has been reported that pathogen induction of the NET-suppressive IL-10 blocks ROS generation [28], consistent with our findings. The balance between inflammation and regulation therefore favors infection persistence and chronicity during *T. spiralis* infections.

Based on the initial description of NETs as an innate effector mechanism [29], studies have focused on various stimuli leading to NETosis and the mechanisms of pathogen evasion from NETs. PMNs stimulated with *Staphylococcus aureus* (*S. aureus*) in vitro exhibited a unique rapid release of NETs, which facilitated the trapping and killing of the pathogen [30]. Neutrophils release NETs in response to nematodes such as *O. ostertagi* and *Haemonchus contortus* [14]. However, other studies have shown that pathogens can escape NET trapping and killing through different strategies. For example, a subset of *S. aureus* captured by NETs continued proliferating after DNase secretion [31]. Studies have also revealed that viruses, such as feline leukemia virus (FeLV) and human immunodeficiency virus-1 (HIV), can not only induce NETosis but can also, in the case of FeLV, target NETs for immune evasion by reducing NET formation through inhibiting PKC activation, subsequently leading to a decrease in ROS production [32, 33]. Besides bacteria and viruses, *Leishmania donovani*, a protozoan parasite, also induces NETs and is trapped by NETs. Still, it can resist microbicidal activity and evade the toxicity of NETs because of its expression of the highly complex glycoconjugate lipophosphoglycan (LPG) [34]. Herein, the inhibition of PMN-derived NET structures by ESP was visualized using fluorescence imaging analyses. We found that the reduction in Ad3-ESP-mediated PMA-induced NET release resulted from a decrease in ROS production rather than from massive cell death. This represents a novel NET evasion strategy employed by *T. spiralis* to aid in evading the host's innate immune response. Qualitative assays revealed inhibition of PMA-induced NET formation and a decrease in ROS production, which is similar to what has been described for Hepatitis B Virus (HBV) proteins [35] and cysteine protease ApdS in *Streptococcus suis* [36]. The reduction of ROS generation may likely prove to be a broad NET-evasion strategy employed by various pathogens [11].

Although NETs play a beneficial role in host defense, an imbalance in NET formation or clearance can lead to autoimmune diseases, such as systemic lupus erythematosus (SLE) and ANCA-associated vasculitis (AAV), by inciting inflammation, causing tissue damage, and exposing self-antigens [37, 38]. In patients with SLE, NETs have a higher number of pathogenic autoantigens compared to the NETs of healthy human PMNs, leading to increased tissue damage [39]. Moreover, NET degradation may be impaired in some lupus patients because of DNase I inhibitors, among other factors, resulting in a more persistent and prolonged NET half-life [40]. On the other hand, autoimmune vasculitis is a chronic condition associated with high concentrations of anti-neutrophil cytoplasmic autoantibodies (ANCAs). The presence of ANCAs targeting major antigens, such as proteinase 3 (PR3) and myeloperoxidase (MPO), suggests that NET-associated components are involved in the pathogenesis of the disease [41]. ANCAs activate PMNs that adhere to endothelial cells, resulting in a local production of ROS and the release of proteolytic enzymes that damage vascular endothelial cells. They also induce NETosis in PMNs, thereby creating a proinflammatory feedback mechanism [42]. Furthermore, various cytokines secreted by activated PMNs are involved in the pathogenesis of AAV, including IL-8, IL-10, IL-1β, and TNF-α. Studies have demonstrated that IL-10 regulates T-cell-mediated immunity, and levels of this cytokine herald a higher risk of AAV relapse [43]. In patients with AAV, inflammatory cytokines (IL-1β and TNF-α) contribute to the priming of PMNs and the upregulation of adhesion molecules on their surface. The capacity of *T. spiralis* ESP to modulate NET production and cytokine secretion suggests that future research should focus on understanding the molecular pathways by which ESP target and inhibit NET formation, which may provide valuable insights into the design of targeted interventions. In addition, identifying the specific active components within ESP responsible for these effects will be crucial for advancing medicine in the context of these diseases.

In summary, the ability of *T. spiralis* ESP to suppress NET production and regulate cytokine secretion offers a promising avenue for developing novel intervention strategies in autoimmune diseases.

### Supplementary Information

**Additional file 1: Figure S1**. Morphology and cell surface markers of CD11b+Ly6G+ neutrophils. (a) Isolated cells from C57BL/6J mice bone marrow were analyzed by flow cytometry for the expression of Ly6G and CD11b (the red selection area represents the purity of up to 81.27% of neutrophils). (b) The morphology of lobulated nuclei of isolated neutrophils was stained by using Hoechst. Scale bars = 40 μm.

## Data Availability

All data generated or analyzed during this study are included in this published article.
