# Supplementary figures and images for: Trichinella spiralis excretory/secretory products from adult worms inhibit NETosis and regulate the production of cytokines from neutrophils
Source: Parasit Vectors. 2023 Oct 20;16:374. doi: 10.1186/s13071-023-05979-8 (PMC10588246; doi:10.1186/s13071-023-05979-8)

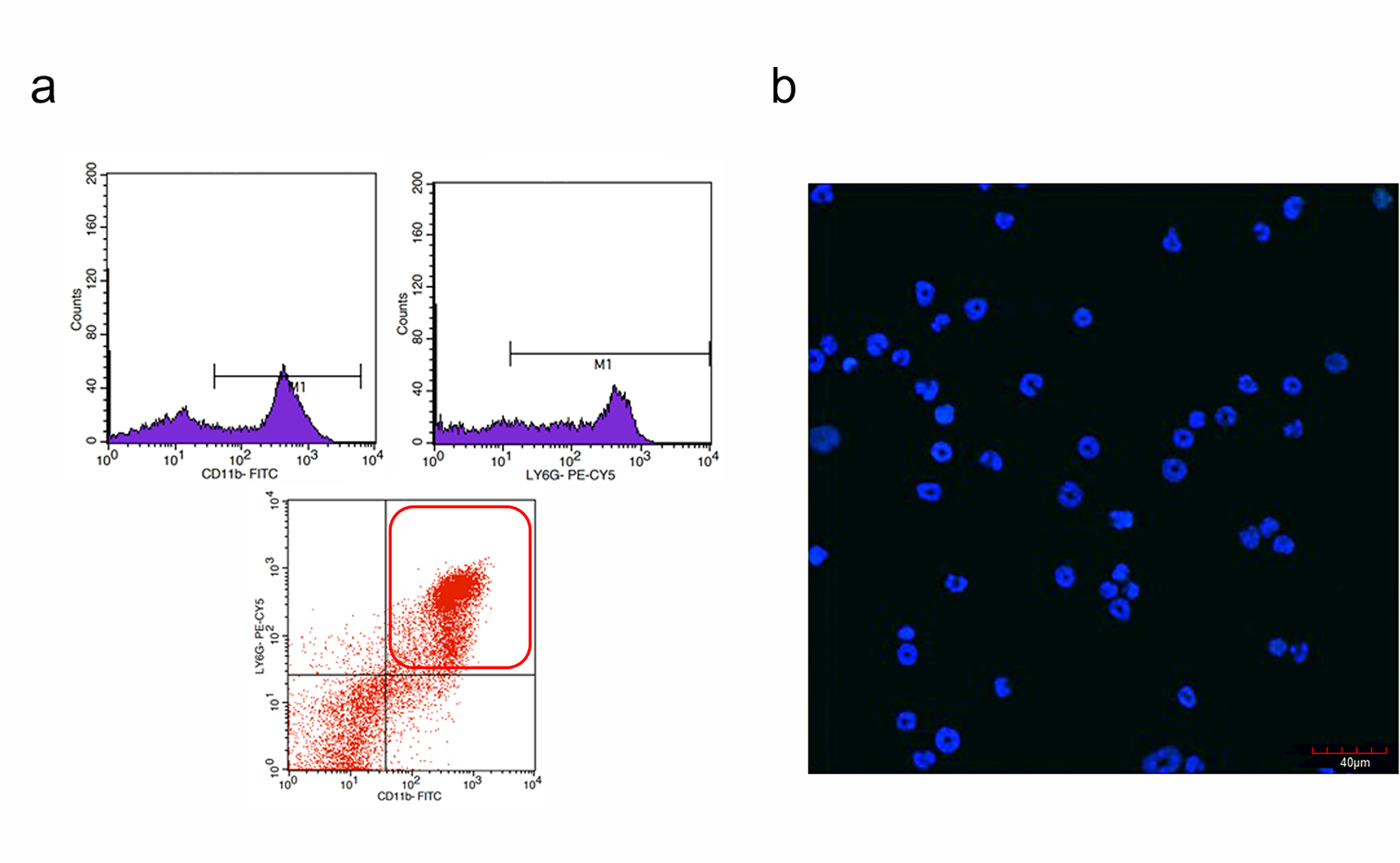

Supplement: Supplementary file 1 — Additional file 1: Figure S1. Morphology and cell surface markers of CD11b+Ly6G+ neutrophils. (a) Isolated cells from C57BL/6J mice bone marrow were analyzed by flow cytometry for the expression of Ly6G and CD11b (the red selection area represents the purity of up to 81.27% of neutrophils). (b) The morphology of lobulated nuclei of isolated neutrophils was stained by using Hoechst. Scale bars = 40 μm. [file 13071_2023_5979_MOESM1_ESM.tif]
